# Modern Views about Consumption

**Published:** 1898-06-11

**Authors:** 


### Modern Views about Consumption.

Our table is covered with a sudden efflorescence of literature from both sides of the Atlantic on consumption and its treatment. What a change has come over the scene since the days when the treatment of phthisis was summed up in the painting of the chest and the administration of cod liver oil! What hopes and possibilities are held before those who can give themselves up to the management of their malady, and how dark, by contrast, the prospect appears to those who must work on and earn their daily bread, even though at the same time they earn an early grave! Indeed, among much that is contradictory and among much that is far from certain, this at least seems clear, that if the man smitten with consumption is to have a reasonably good chance of recovery the purse-strings must be unloosed and money must be spent with a free hand.

The key-note of the modern treatment of consumption hinges on the idea that the disease is to a large extent the outcome of a man's surroundings, and that unless these are altered the disease will go on. Thus it is that we find such stress laid upon climate and change of climate. We may, indeed, fairly suppose that a good deal of the greater hopefulness in regard to the prognosis of consumption, which is so striking a feature in the writings of physicians on the subject in these latter days, is due to the increased facilities which now exist for obtaining change without that exhaustion and that risk which travel used to involve in former times. Thus do the hotel-keepers flourish. There are difficulties, however, about hotels. On the one hand, ordinary travellers are becoming afraid of mixing with consumptives, and on the other the consumptives are being taught that the good of travel lies in the fresh air, which they very obviously do not get in the hotels. Life in a special sanatorium for consumptives may not be altogether a joy, but it seems probable that the more thoroughly people come to appreciate the exact nature of their disease, the more they will drift into special institutions in which all the arrangements are specially adapted to the treatment of their ailment.

In the establishment of such sanatoria we see prospect of some hope for those less fortunate ones to whom money is an object of much moment, for, if we take the bare essentials, a sanatorium for consumptives ought not to be an inordinately expensive establishment. Fresh air is cheap; cleanliness need not be expensive when walls and floors are bare; and in any case man must eat, whether he be in a sanatorium or not. One can, indeed, hardly doubt that if once the sanatorium system were fairly introduced, and if establishments were set up on several scales to suit different purses, it would at last be possible to obtain proper treatment for the early stages of consumption, which at present is not the case. Notwithstanding the enormous prevalence of phthisis in England, we have no hesitation in saying that practically none but the wealthy are able to obtain proper treatment in its early stages. A small select few, after much waiting for their turn, can be received at Ventnor and one or two other places, where they will be well dealt with; but for the great mass no provision is made until they get "ill"—and the consumptive in the early and curable stage of his complaint is not what the British workman would call "ill." What, then, about medicine? Is medicine to do nothing? Sir Samuel Wilks says, "The only remedies I know for consumption are air and sunshine—Air, Air, FRESH AIR"; and the special tuberculosis number of the *Practitioner*, a volume of 155 pages, containing the latest views of many leading authorities, gives but nine pages to the medicinal treatment of the disease! Of the many methods which have been put forward for the drug treatment of consumption, the free use of creasote or of guaiacol, the saturating of the patient with these or equivalent drugs, seems at the present moment to be the course which is supported by the most trustworthy evidence. We fear, however, that it must be admitted that, notwithstanding the number of drugs which have been vaunted as having a germicidal power over the bacilli of tuberculosis, we are so far without any specific remedy for the disease. A good deal, however, can be done, even while a patient is living in his own home and carrying on his own work, to increase his resisting power, so as to put him in the best condition to withstand the inroads of the disease. This, which we may call the management of phthisis, as apart from its cure, is a large subject, and the drugs which are useful are many, but a careful perusal of the pile of tuberculosis literature before us only serves to strengthen and drive home the conviction, on the one hand, that the death-rate from tuberculosis can be lowered far more effectually by efforts at prevention than by treatment of whatever sort; and on the other that, where prevention fails, the best hope lies, as Sir Samuel Wilks expresses it, in "air and sunshine—Air, Air, FRESH AIR," whether these be got in high mountains, amid snow and ice, or on the sun-baked desert, whether on the veld or on the sea, or even in our own land in special institutions.

